# Protobiotic Systems Chemistry Analyzed by Molecular Dynamics

**DOI:** 10.3390/life9020038

**Published:** 2019-05-10

**Authors:** Amit Kahana, Doron Lancet

**Affiliations:** Dept. Molecular Genetics, The Weizmann Institute of Science, Rehovot 7610010, Israel; amitmiti@gmail.com

**Keywords:** systems chemistry, systems protobiology, molecular dynamics, gard, lipid world, micelle, origin of life

## Abstract

Systems chemistry has been a key component of origin of life research, invoking models of life’s inception based on evolving molecular networks. One such model is the graded autocatalysis replication domain (GARD) formalism embodied in a lipid world scenario, which offers rigorous computer simulation based on defined chemical kinetics equations. GARD suggests that the first pre-RNA life-like entities could have been homeostatically-growing assemblies of amphiphiles, undergoing compositional replication and mutations, as well as rudimentary selection and evolution. Recent progress in molecular dynamics has provided an experimental tool to study complex biological phenomena such as protein folding, ligand-receptor interactions, and micellar formation, growth, and fission. The detailed molecular definition of GARD and its inter-molecular catalytic interactions make it highly compatible with molecular dynamics analyses. We present a roadmap for simulating GARD’s kinetic and thermodynamic behavior using various molecular dynamics methodologies. We review different approaches for testing the validity of the GARD model by following micellar accretion and fission events and examining compositional changes over time. Near-future computational advances could provide empirical delineation for further system complexification, from simple compositional non-covalent assemblies towards more life-like protocellular entities with covalent chemistry that underlies metabolism and genetic encoding.

## 1. Systems Chemistry in Life’s Origin

In his last paper, Alexander Oparin, life’s origin pioneer, wrote: “The process of evolution of organic compounds that led to the emergence of life can be divided into two major stages: chemical and prebiological. Chemical evolution developed at the molecular level, obeying chemical laws to reach abiogenic synthesis of polymers that resulted in a spontaneous assembly of phase-separated thermodynamically open systems, or probionts. The appearance of probionts was accompanied by the development of a new law they obeyed, i.e., natural Selection” [1]. Translated to more modern lingo, life’s origin began with abiogenic processes, generating the chemical components needed for life’s emergence. This provided the infrastructure for the emergence, in a later stage, of the first primitive life forms, which could be further complexified by rudimentary evolution. In line with NASA’s definition of life [2], the second stage must have involved the advent of some scheme of reproduction. This is reflected in Freeman Dyson’s words: “As soon as the garbage-bag world begins with crudely reproducing protocells, natural selection will operate to improve the quality of the catalysts and the accuracy of the reproduction” [3].

What Oparin called probiont, also termed protobiont or protocell, is defined by the Oxford dictionary as “An entity consisting of a small drop of aqueous solution surrounded by a membrane and containing complex organic molecules, hypothesized as ancestral to living cells”. Evidently, Oparin alludes to the idea that the transition from abiotic entities to such capable of natural selection was not centered on a single molecule, but rather constituted a multicomponent system. In this respect, Oparin may be legitimately considered as a pioneer of systems chemistry and its relationship to the origin of life. This distinction between what single molecules can do and what necessitates a molecular ensemble is indeed reflected in the general definition of systems chemistry. This field seeks insight into complex networks of interacting molecules deriving systems-level characteristics that emerge through collective behavior [4].

Notably, from its inception, systems chemistry has been tightly linked to studies on the origin of life, and is sometimes thought of as focusing on the origin of replication at the molecular level [5]. Some of the roots of this field rest, for example, in “research on the autocatalytic self-replication of biological macromolecules, first of all of synthetic deoxyribonucleic acids” [6]. Other domains of the field include colloidal particles, soft droplets, and nanocrystals, [7], reminiscent of Oparin’s coacervates. A diversity of facets of this field of chemistry interestingly echoes the variety of chemical models for life’s origin.

An appreciable sector of life’s origins models adheres to the credo of systems chemistry, asserting that life likely began as a multi-molecular system capable of reproduction, selection, and evolution. Along these lines, early abiogenesis was followed by systems protobiology [8], a stage at which an assemblage of chemical compounds began to acquire life-like systems properties. In the eyes of many, this must have involved autocatalytic sets or mutually catalytic networks, as insightfully stated by the founders of systems chemistry: “autocatalytic feedback is the least common denominator of all scientific theories dealing with the origin of life, regardless of whether the core is genetic, metabolic or containment-based; (it is thus) highly desirable to focus on research dealing with autocatalytic systems” [5]. The theoretical and experimental infrastructure for this view has been in existence for decades prior to the nominal advent of the Systems Chemistry concept [9,10,11,12,13,14], as reviewed [8,15,16].

## 2. Molecular Dynamics for Systems Chemistry

Molecular Dynamics simulation was first developed in the late 1970s [17,18], awarding Levitt, Warshal, and Karplus the 2013 Nobel Prize in Chemistry [19]. The method entails a numerical solution of the classical Newtonian equations of motion for a group of atoms. This necessitates defining the laws that govern the interactions between atoms, combined with the atomic positions that provide the associated potential energy and the forces on the atoms. The laws are approximated with different degrees of realism using a variety of physical methodologies [20]. Given the initial positions and velocities for every modelled atom in the system, the molecular dynamics algorithm computes the time progression of the atomic coordinates and momenta, i.e. the time-dependent dynamics of the atoms and molecules involved [21].

Over the past four decades, molecular dynamics has advanced from simulating several hundreds of atoms to systems having 50,000–500,000 atoms [22]. This encompasses entire proteins in water solution, membranes with their embedded proteins, as well as large macromolecular complexes such as RNA polymerase [23], nucleosomes [24,25], and ribosomal subunits [26,27]. In parallel, reaction networks related to self-replication in autocatalytic networks have been studied by molecular dynamics [28]. This progress is mainly due to advances in high performance computing, affording simulations of large multi-molecular systems at high resolution (atomistic representation or all atoms). In parallel, united-atom and coarse grained representations have been used for more complex molecular ensembles and/or when long-time simulations are required [29,30].

Molecular dynamics simulations have thus evolved into a mature technique that can help effectively understand molecular and macromolecular structure and dynamics, as reviewed [21,22]. This includes the patterns, strength, and characteristics of molecular interactions, as well as the conformational changes that molecules may undergo. Hence, molecular dynamics is now widely used in the analysis of ligand-receptor interactions [31], protein folding [32], enzyme action [33], and lipid assembly dynamics [34]. We note that a majority of such simulations aim for portraying only non-covalent reactions, such as in ligand-receptor docking, micelle formation, and protein folding.

In the past, experimentation and theory of molecular interactions and dynamics were distinct entities. Theoretical models were usually depicted in a set of thermodynamic and kinetic equations leading to overall numerical predictions. The validation of such models depended on their ability to predict the experimental results [21]. A severe drawback to most models was that their equations could rarely represent the full complexity of the real-world problems, and a considerable, sometime insurmountable amount of experimental simplification was necessary [35]. Thus, some years ago, theoretical models could be tested only in special circumstances, and many scientific problems could not be easily modeled.

The recent massive improvement of high-speed computing led to the introduction of computer experiments, a new mediator between experiment and theory, substantially modifying the traditional chasm between theory and experiment. Computing power now allows more realistic systems to be modeled, leading to a better understanding of real-life experiments [21]. Further, effective molecular dynamics simulations demand better models, affording the improvement of the theory. At present, the computer experiment results can be compared directly and accurately with laboratory experimental results, even in appreciably complex systems. This makes molecular dynamics simulation a potent tool, not only for comprehending and interpreting the experiments, but also in examining domains inaccessible to experimentation [36]. Not less importantly, molecular dynamics results may suggest new experimental paths, thus enhancing scientific creativity [21].

Systems chemistry is already leveraging the above-mentioned revolution. In one example [37], molecular dynamics simulations were used to probe the structural details of self-assembling, self-replicating molecules, and compute their electromagnetic absorption spectra. Two proposed structural models were then tested by comparing the calculated spectra to experimental data, thus elucidating the role of peptide β-sheet formation and aromatic ring stacking in the stability of the self-assembled fibers. In another example [38], the authors developed a systems chemistry approach assisted by molecular dynamics to design allosteric synthetic receptors and their cognate ligands using a dynamic combinatorial strategy. Analysis of the stepwise formation of the complex indicates that binding of two partners by the central macrocycle exhibits significant positive cooperativity, as expected for an allosteric system.

## 3. GARD: A Lipid-Based Systems Chemistry Model for Life’s Origin

The graded autocatalysis replication domain (GARD) model belongs to the mutually catalytic networks approach to life’s origin, specifically embodied in a lipid world scenario [8,11,39]. GARD has been shown by computer simulations of realistic chemical kinetics equations to portray replication/reproduction behavior mediated by homeostatic growth [40], as well as selection and evolution [41]. Its quasi-stationary states in compositional space, capable of reproduction-like behavior, have been termed composomes. GARD’s strong point is in invoking supramolecular structures that are both replicable and evolvable, as well as being simple enough to be simulated by detailed molecular dynamics.

A known deficiency of GARD is the paucity of experimental verification of many of its predictions. Such experimental evidence should address the question of whether lipid assemblies, e.g., micelles, are capable of homeostatic growth and even rudimentary transfer of compositional information to fission-generated progeny. These experiments would require complex setups and accurate compositional monitoring of individual microscopic amphiphile assemblies, currently mostly outside the realm of experimental scrutiny. Some promising leads do however exist in the recent experimental exploration of multi-component lipid vesicles [42,43,44,45]. Another critique of GARD asserts that it simulates abstract molecules without specified chemical properties. This point has been recently addressed in an extension of the simulated model to incorporate realistic physicochemical properties of amphiphilic molecules, showing that a measure of compositional heredity may be observed. However, such a study is still quite remote from a detailed inspection of the behavior of individual molecules.

The prospect of full-fledged molecular dynamics simulations of the GARD model could provide an important supplement to the expected laboratory experimental evidence. This would specifically generate a path to analyzing the dynamic behavior of complex molecular mixtures, e.g., in heterogeneous lipid micelles, utilizing specific models for molecularly realistic amphiphiles. Lipid micelles are particularly attractive as a platform for GARD molecular dynamics analyses, because their smaller size (a few hundred molecules) is more compatible with the emergence of homeostatic growth [46], when compared to the smallest vesicles with about a million molecules. Further, there is considerable literature coverage of micellar molecular dynamic simulations, including cross-validation with experiments, as addressed in the next chapter.

## 4. Molecular Dynamics of Micelles and Mixed Micelles

Amphiphilic molecules are endowed with the capacity to self-assemble and generate supramolecular structures. The possibility to obtain relevant information on the emerging structures, compositions, and energy parameters, as well as the capacity to model and predict the self-organizing mechanisms of such molecules, was augmented by the advent of computational chemistry. This prominently includes molecular dynamics analyses, initially coarse-grained and more recently increasingly accurate scrutiny, which allows for better understanding of amphiphile dispersion, self-assembly, and segregation.

The history of using molecular dynamics for understanding micelles of lipids and surfactants dates back >30 years [47,48,49], with dozens of papers published in the first half of this period [50,51,52,53,54]. This attests to scientific and technological maturity that ensures diverse analytic capacities. Many of the early studies were performed with surfactants, such as sodium dodecyl sulfate [54,55], and simple lipids, such as lysophosphatidylethanolamine [47], but the utilized amphiphile repertoire later diversified to include molecules, such as those forming ionic liquids [56,57,58] and those with complex headgroups, such as alkyl-polyglycolethers [59]. Studies included the effect of counter-ions, hydrophobic side chain mobility, and interactions with other molecules, as reviewed [60]. Relevant to mutually catalytic networks embodied in amphiphilic micelles (next chapter) are molecular dynamics studies that examine the kinetics and energetics of micelle-related monomer association [61] and dissociation [62,63], as well as solute partitioning into micelles [64]. Likewise, the proposed capacity of micelles to undergo fission is supported by studies on the influence of micelle constituents on micellar morphology, stability, deformation, and disruption [65,66,67]. In the abovementioned studies, the in silico results are often compared with experimental information as exemplified [68], providing validation to the computational approaches.

One study [69] implemented coarse-grained accelerated molecular dynamics to study aqueous solutions of seven different nonionic polyethylene glycol (PEG) surfactants, encompassing about a million atoms. In the realm of thermodynamics, this allowed calculating the critical micelle concentrations (CMCs) as a function of the length of the hydrophobic tails and PEG head groups, showing good agreement with experimental data. The researchers also characterized the size and shape of such micelles. In the kinetic realm, they further observed that the micelles composed of relatively hydrophobic surfactants continue to grow beyond the nominal simulated duration. This suggested that the equilibrium micelle size required longer simulations or advanced sampling techniques, so as to predict the properties of slowly evolving surfactant systems.

Another study [70] aimed to illustrate the sources contributing to the entropy increase in micellization. Explanations concerning the structure of water molecules surrounding micelles and the relative freedom of hydrocarbon chains were offered but proved hard to evaluate experimentally. By using molecular dynamics, the authors were able to quantitatively distinguish between changes in translational, rotational, and vibrational entropy of each sodium dodecyl sulfate (SDS) amphiphile in an assembly, differentiating the distinct contributions of hydrocarbon tails and hydrophilic head groups. A similar study [63] examined the escape of amphiphiles from simulated ionic and nonionic micelles by directly measuring escape kinetics and potentials of mean force along the escape coordinate. In this study, different force field algorithms of both coarse-grained and atomistic models were employed and evaluated by comparison to experimental results. Both studies represent the utility of molecular dynamics in deriving physicochemical parameters from simulated micelles and their environments, while also detecting and analyzing kinetic events, such as micellar exit and entry of amphiphiles [71]. In the same vein, micellar structural transformations may also be studied [67].

Mixed micelles, colloidal particles, and nanoparticles have attracted considerable attention, including in the realm of drug delivery. Recently, this field has been boosted by the use of molecular dynamics, in conjunction with other computational methods [72], leading to insight on how theoretical concepts and computational models can predict different experimentally measured physicochemical properties of self-aggregation processes of mixed molecular systems. One relevant study [73] aimed to research micelles composed of different compounds (mixed micelles) so as to determine the self-assembly behavior of pure Brij35 (polyethylene glycol dodecyl ether) and its admixtures either with CTAB (cetyltrimethyl ammonium bromide) or with SDS (sodium dodecyl sulfate) at different concentrations. The structure and composition of the computed mixed micelle were then shown to affect the partition equilibria of various extraneous solute molecules, mostly lipophilic, and the results were in good agreement with experimental data.

Other studies probe the synergistic effects of components within mixed amphiphiles, with direct relevance to the potential prebiotic micellar behavior. For example, [59] studied the interfacial tension of emulsions with mixed surfactants in aqueous solutions by employing coarse-grained molecular dynamics models, with validation by tensiometry experiments. The authors discovered that mixing resulted in a significantly lower interfacial tension. This synergistic effect is proposed to occur due to closer headgroup packing, a phenomenon that could be manifested also on micellar surfaces and play important roles in micelle accretion and lipid exchange.

The foregoing micellar renderings address only non-covalent transformations, such as lipid entry and exit, as well as micelle nucleation growth and fission. These are directly relevant to the inner works of the basic GARD model, which invokes mutually catalyzed lipid accretion. Such relevance is underlined by molecular dynamics studies of life’s origin models that invoke lipid involvement. One study reported the suitability of lipid phases for heterogeneous catalysis, i.e. the increase of reaction rates at the interface [74]. Another simulation study offers a model of prebiotic self-replication of lipid assemblies driven by environmental factors [75]. A third study addresses the phenomenon of enhanced concentration of encapsulated proteins during vesicle formation, relevant to prebiotic compartmentalization. They analyze bilayer-vesicle transition by molecular dynamics, and report that "bilayer bulging" leads, under some conditions, to enhanced protein encapsulation [76]. All the forgoing reports portray the relevance of molecular dynamics as an arena for computer experiment validation of non-covalent lipid-based prebiotic models.

In parallel, for several decades, research has been done on the capacity of lipid micelles and vesicles to also exert covalent catalysis in enhancing the rate of amphiphile production from chemical precursors [77,78]. Such reactions would be a challenging target for molecular dynamics simulations. Similar covalent reactions are at the heart of the Metabolic GARD model we have recently proposed [8]. Such forward-looking progress would help understand mechanisms that are relevant to early life’s evolutionary progression, including metabolism, enzyme catalysis, and polynucleotide replication.

## 5. Roadmap for GARD Evidence via Molecular Dynamics

Performing GARD model simulations with molecular dynamics requires crucial proficiencies. Thus, the simulation must be able to accurately recognize micellar assemblies and ascribe each molecule either to a micelle or to the surrounding simulated environment as a free monomer. This could be achieved by calculating local centers of mass [30] or, more commonly, clustering molecular components together and assigning them to the same aggregate based on spatial distances, often by setting a limiting threshold [71,79]. By tracking these micelles along the experiment timeline, compositional vectors for each micellar state could be defined. Since micellar fission occurs in the 10 nanosecond time range, it is compatible with molecular dynamics [80,81,82], hence fission events could be discerned and analyzed as described [75,82]. Likewise, fusion events, despite being relatively rare, could be analyzed [83].

Regarding system size, if a simulation can contain about 500,000 atoms [22], it could accommodate 10,000 lipids with a typical atom count of 50. At the lower bound, 1000 lipid molecules will be simulated, whereby 50,000 atoms are lipids. Even 1000 lipid molecules are sufficient for GARD simulations, since with a micelle size of 50–200 amphiphiles, this allows one to simulate 5–20 micelles, and if the GARD mixed micelle has ten types of lipids, there would be 5–20 molecules of each type. Such molecule and atom counts are also compatible with fission, which requires pre-fission micelle elongation by a factor of about 2 to become unstable and undergo fission [80].

The GARD model is opportunistic, affording the participation of diverse amphiphile types [8], and thus can be initially tested by utilizing already existing atomic models for certain amphiphilic molecules. Moreover, some of these molecules have already been proven to behave in accordance with experimental data, even when featured in coarse grained molecular dynamics simulations, harnessing variations of the well-studied lipid-oriented MARTINI force field [79]. This could indicate a possibility for accurate simulations of the GARD model, even in coarse grained resolution.

Applying molecular dynamics to GARD allows one to take advantage of its existing molecular definition and detailed kinetic equations [11,41,84,85]. This is in contrast to more general models (e.g., [86,87]) that are less readily simulatable by molecular dynamics.

We envisage two major approaches to the provision of molecular dynamics evidence for the validity of the GARD model. The first approach to be explored involves full atomistic simulations of individual micelles, as described in [34], focusing on their formation and initial growth. This approach is becoming realistic in view of recent progress in molecular dynamics simulations of relatively complex structures, with up to 500,000 atoms [22]. We intend to ask specific questions on the capacity of heterogeneous lipid micelles to portray compositional preservation upon growth by monomer accretion [67]. Among others, we propose to follow transitions from a premicellar state, involving a small number of loosely bound monomers [69,88] to full-fledged micelles. The proposed simulations will be typically performed with a repertoire of 5–20 types of micelle-generating lipids.

The second approach to be explored involves a relatively recent development in the field, employing Markov state modeling (MSM) methods [89,90]. In this approach, many short molecular dynamics simulations define the underlying energy landscape, allowing both thermodynamic and kinetic constants to be inferred [90]. In the case of GARD, kinetic and catalytic constants for the entry and exit of amphiphiles [40,91] can be obtained. This allows one to enhance the chemical realism of the individual rate constant, as compared to the currently used statistical allocations [11], based on a nature-like distribution [92]. As prescribed for the MSM methodology, the molecular dynamics-based constants can then be plugged into the existing Monte Carlo GARD simulations [11], providing crucial experiment-like support for the capacity of GARD assemblies to undergo reproduction.

An advantage of the MSM method is related to the fact that all atom molecular dynamics simulations for long time-scales may be limited by computational resources. Such a problem may be alleviated by clever MSM sampling techniques, whereby many independent simulations can be easily parallelized. This can be done by using the molecular dynamics simulation software for large systems (MODYLAS), a highly parallelized general-purpose program with scalable fine-grained algorithms [93,94]. Such parallel simulation schemes may efficiently harness available computational resources, as used by the Folding@home endeavor, with 100,000 personal computers used for massively distributed molecular dynamics simulations of protein folding [95].

**Figure 1:**
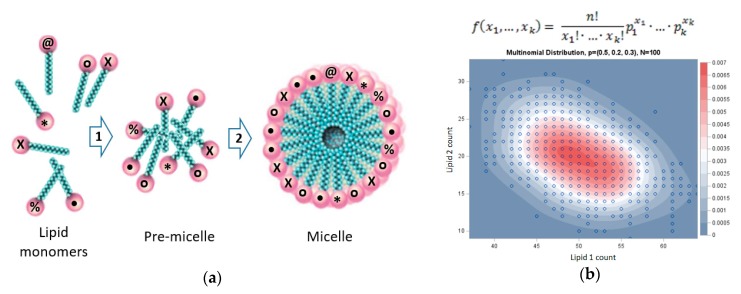
Micelle dynamics and statistics. (**a**) Accretion of lipid micelle with a premicellar intermediate. Shown is a lipid repertoire with a diversity of N_G_ = 6, a premicelle with n = 8 monomers, and a micelle with n = 21 monomers (numbers are for illustration only, see Table 1). (**b**) The multinomial distribution statistics of mixed micelle dynamics. Shown is an example statistic for n = 100 and three lipid types and equal Pi values (Table 1). Premicelle is nucleated at random with a relatively high probability f = 3.0 × 10^−3^. The micelle is illustrated as growing by a certain degree of homeostatic growth, to be assessed by compositional correlation, from the premicelles, reaching a low-probability composition with f = 5.6 × 10^−5^. Importantly, beyond the transition to low probability composition, which signifies an entropy decrease upon growth, there is a clear sign of homeostatic growth manifested in the high normalized dot product H = 0.98 between the premicelles and the fully grown micelle (see [11]), as compared to H = 0.72 ± 0.04 for randomized compositional vectors for the micelle. Figure modified from: https://blogs.sas.com/content/iml/2013/08/05/simulate-from-multinomial-distribution.html and multinomial distribution formula from: http://www.real-statistics.com/binomial-and-related-distributions/multinomial-distribution/.

The molecular dynamics simulations will follow the kinetics of micellar growth, e.g., from premicelles to micelle, seeking evidence for the following two predicted phenomena:

1. With the environment being equimolar in the different lipid types (Table 1), we will look for statistically significant deviations from randomly disposed within-micelle equimolarity. It will be necessary to distinguish the contributions of equilibrium-related statistical deviations stemming from kinetic (rate-enhancement) effects, as expected in the GARD model [11]. One possible test for the relative contribution of kinetics and thermodynamics to compositional biases would be a comparison along the accretion process to asymptotically long simulation times, whereby the micellar composition is likely to be governed purely by thermodynamic equilibrium constants. Spatial deviations from randomity (akin to lipid rafts and caveoli in living cells [91,96]) would also point to thermodynamic effects.
2. We will examine whether compositional fluctuations are amplified by the anticipated mutually catalytic effects, portraying attractor phenomenology (Figure 1). Such attractor behavior is manifested in a progression whereby random initial fluctuations are augmented by the acting mutually catalytic network towards the emergence of a reproducing composome [8]. We are currently studying such behavior by standard kinetic Monte Carlo simulations to pave the way for future molecular dynamics scrutiny.
3. We will examine the net incoming time-dependent amphiphile fluxes for a correlation of their compositional direction to that of the initial premicelles. Such correlation will provide evidence of compositional preservation, the hallmark of homeostatic growth, and an equivalent to compositional replication/reproduction [8].
4. We will seek simulated fission events affecting the biasedly grown micelles [80], showing better-than-random similarity between the parental micelle and its progeny.

In addition to the aforementioned molecular dynamics simulation plans for the standard GARD model, whereby all catalysis event are non-covalent, we should lay the foundations for analyzing the behavior of the new metabolic GARD model [8,97]. For this, molecular dynamics versions capable of simulating the formation and breakage of covalent bonds as described in [23,98,99] is essential. In a typical example, covalent non-enzymatic catalysis is exerted by one lipid molecule in a lipid assembly, enhancing the rate of covalent modification of the head group of a neighboring lipid as described in the realm of the GARD model [84], as well as experimentally [45,78]. In a full-fledged metabolic GARD simulation, a complete mutually catalytic network of such covalent chemistry can be simulated, as described [28].

## 6. Conclusions

The GARD model for life’s origin is based on the notion that the first rudiments of life were not individual molecular replicators, but rather self-reproducing multi-molecular systems, a concept pioneered by Oparin [1]. As reproduction capacities emerged, systems chemistry seeded a long evolutionary chemistry process which culminated in entities analyzable by systems biology. We have offered the term “systems protobiology” for the intermediate stages, including the possible early emergence of heterogeneous lipid assemblies, governed by GARD dynamics, and a capacity to gradually lead to other aspects of present-day life [8].

Among the strengths of the GARD model are its realistic depiction of the emergence of life-like entities from a prebiotic mixture of random chemicals [100], and its capacity to underlie a concrete molecular basis for molecular dynamics simulations. The foregoing roadmap is intended to indicate some of the possible computer experiments that could attempt to confirm the dynamic non-equilibrium features of the GARD model, which underlie the emergence of lipid-based compositional replicators. This should enhance the model’s realism, whereby, rather than being based on statistically-derived catalytic properties, the newly simulated GARD will be grounded in bona fide atomically-detailed molecules.

There are severe limitations in providing laboratory evidence for GARD. This is because a mutually catalytic micellar lipid network is expected to initially involve weak, relatively low-specificity interactions, and hence would likely portray low fidelity compositional reproduction. Further, any process in which the fidelity might gradually improve would require long-term evolutionary chemistry, suggesting the necessity for very long-term laboratory experiments. In this respect, molecular computerized experiments could be the only realistic path for the validation of GARD.

Given the riches of the presently available molecular dynamics capabilities, it is likely that such proposed initial steps will serve as roots for a much more detailed scrutiny. The concrete predictions for the increased capacities of the methodology over the next two decades [98], potentially enhanced by quantum computing [101], suggest a measure of optimism regarding a full-fledged computerized emulation of compositional reproduction of lipid assemblies, and possibly a gradual evolutionary systems protobiology experimentation portraying the transition from such simple assemblies towards more elaborate protocells.

## Figures and Tables

**Table 1:** Multinomial distribution statistics of lipid accretion and homeostatic growth.

Lipid Type	%	•	X	*	@	o	
X_i_	X_1_	X_2_	X_3_	X_4_	X_5_	X_6_	n
P_i_	0.167	0.167	0.167	0.167	0.167	0.167	
Premicelle X_i_	1	2	2	1	0	2	8
Micelle X_i_	2	6	5	2	1	5	21
